# Quantifying the hepatotoxic risk of alcohol consumption in patients with rheumatoid arthritis taking methotrexate

**DOI:** 10.1136/annrheumdis-2016-210629

**Published:** 2017-03-23

**Authors:** Jenny H Humphreys, Alexander Warner, Ruth Costello, Mark Lunt, Suzanne M M Verstappen, William G Dixon

**Affiliations:** 1 Arthritis Research UK Centre for Epidemiology, Manchester Academic Health Science Centre, University of Manchester, Manchester, UK; 2 NIHR Manchester Musculoskeletal Biomedical Research Unit, Central Manchester Foundation Trust, Manchester Academic Health Science Centre, Manchester, UK; 3 Health eResearch Centre, Farr Institute, Manchester Academic Health Science Centre, University of Manchester, Manchester, UK

**Keywords:** Rheumatoid Arthritis, Methotrexate, Epidemiology, Treatment

## Abstract

**Background:**

Patients with rheumatoid arthritis (RA) who take methotrexate (MTX) are advised to limit their alcohol intake due to potential combined hepatotoxicity. However, data are limited to support this. The aim of this study was to quantify the risk of developing abnormal liver blood tests at different levels of alcohol consumption, using routinely collected data from primary care.

**Methods:**

Patients with RA in the Clinical Practice Research Datalink starting MTX between 1987 and 2016 were included. Hepatotoxicity was defined as transaminitis: alanine transaminase or aspartate aminotransferase more than three times the upper limit of normal. Crude rates of transaminitis were calculated per 1000 person-years, categorised by weekly alcohol consumption in units. Cox proportional hazard models tested the association between alcohol consumption and transaminitis univariately, then age and gender adjusted.

**Results:**

11 839 patients were included, with 530 episodes of transaminitis occurring in 47 090 person-years follow-up. Increased weekly alcohol consumption as a continuous variable was associated with increased risk of transaminitis, adjusted HR (95% CI) per unit consumed 1.01 (1.00 to 1.02); consuming between 15 and 21 units was associated with a possible increased risk of hepatotoxicity, while drinking >21 units per week significantly increased rates of transaminitis, adjusted HR (95% CI) 1.85 (1.17 to 2.93).

**Conclusions:**

Weekly alcohol consumption of <14 units per week does not appear to be associated with an increased risk of transaminitis.

## Background

Methotrexate (MTX) is the first-line disease-modifying antirheumatic drug in patients with rheumatoid arthritis (RA).^1^ It is clinically effective and well tolerated;^2^ however, the potential hepatotoxicity of MTX remains a concern,^3^ and regular blood monitoring is mandated. Alcohol consumption is also well known to have an adverse effect on the liver, particularly in excess.^4^ ^5^ Given these two associations, patients taking MTX have traditionally been advised to limit or even abstain from alcohol consumption. The American College of Rheumatology (ACR) guidelines, published in 1994, recommend abstinence from alcohol with only occasional exceptions.^6^ In contrast, more recent guidance from the British Society for Rheumatology, published in 2008, suggests that patients taking MTX should limit their alcohol intake to ‘well within the UK national recommendations’,^7^ without further specification.

While the relationship between MTX and hepatotoxicity has been extensively reviewed,^3^ there is a lack of evidence to quantify the potential additional effect of alcohol on liver toxicity while taking MTX. Indeed, the ACR guidance comments that regular alcohol consumption should not occur since there are ‘no data about the quantity of alcohol that can safely be consumed with MTX’.^6^ The majority of studies which have examined this question have focused on histopathological changes in serial liver biopsies,^8^ ^9^ and frequently date back to the 1970s. By contrast, current monitoring guidelines advocate measuring of serum liver function tests (LFTs).^7^ Importantly, previous studies have not consistently demonstrated an association between increased alcohol consumption and hepatotoxicity or liver damage;^9–14^ yet, it is clearly biologically plausible that there may be an additive. Many patients would like to drink modestly; in the absence of evidence, such patients may be inclined or advised to either abstain from alcohol altogether or avoid MTX, a potentially beneficial drug. If patients do drink alcohol alongside MTX, even in moderation, they anecdotally describe feeling anxious or ill at ease. Understanding whether there is a safe amount of alcohol that can be consumed alongside MTX, and what that amount is, would significantly aid informed decision-making.

The aim of this study, therefore, was to quantify the risk of alcohol consumption on hepatotoxicity in a contemporary group of MTX users with RA, in a large national primary care database.

## Methods

### Patients and setting

Patients with RA within the Clinical Practice Research Datalink (CPRD) were identified using a previously validated algorithm.^15^ CPRD is a large electronic database of routinely collected primary care electronic medical records, beginning in 1987, which includes approximately 8% of the total UK population and is considered broadly representative of the UK population in terms of age, gender and ethnicity.^16^ In the UK, MTX therapy is typically initiated in a secondary care setting by a rheumatologist, but subsequent prescriptions and blood monitoring are performed in primary care, and are therefore recorded within their primary care electronic records. All patients with RA starting MTX after 1987 were included once a practice had met data quality standards required for participation in CPRD. Follow-up was commenced from the date of the first MTX prescription and continued until February 2016, unless patients were censored earlier (see below).

### Exposures and outcome

The outcome of interest was an episode of transaminitis, defined as alanine transaminase (ALT) or aspartate aminotransferase (AST) levels of three times the upper limit of normal (ULN) or higher, according to local laboratory standards. Patients were included in the analysis if they had ALT and AST measured on average at least six times per 12 months to indicate compliance with regular blood monitoring and avoid introducing surveillance bias. Prior studies have identified persistently raised LFTs as being predictive of progression to cirrhosis;^17^ hence we had a secondary definition of transaminitis as three sequential ALT or AST measurements above the ULN. Alcohol consumption was identified first as yes/no, then by units of alcohol consumed per week. A unit of alcohol represents 10 mL or 8 g of pure alcohol,^18^ and is used in the UK to make comparisons of alcohol consumption across different beverages. It is also used by the UK government to set national guidelines; currently, the guidance is to drink no more than 14 units of alcohol per week for both men and women.^19^ Prior to January 2016, the limit for men was higher at 21 units per week. For patients who had alcohol status recorded more than once within CPRD, the value used was the earliest recorded alcohol consumption data following first MTX prescription. If this was not available, then the nearest alcohol consumption data recorded prior to the first MTX prescription were used. If the only data available on alcohol consumption was yes/no, patients who did not drink were recorded as drinking zero alcohol units, to increase the power of the study. As patients sometimes undertake pauses in their MTX treatment, either through their own choice or through clinician recommendation, person-time was included in the analysis only while patients were actively receiving MTX. Thus, person-time and events of transaminitis occurring while the patient was not taking the drug were excluded. Patients were censored at the time of the first episode of transaminitis, death or 29 February 2016.

### Statistical analysis

Crude rates of transaminitis were calculated per 1000 person-years first for all patients, then in drinkers versus non-drinkers and finally by dividing alcohol units into categories of increasing consumption (0/1–7 (mild)/8–14 (moderate)/15–21 (moderate–high) and >21 (high)). Cox proportional hazard models were used to investigate the association between alcohol consumption and time to first episode of transaminitis, both univariately and age and gender adjusted. As for the crude rates, a number of different models were constructed. First, the risk of transaminitis was identified in drinkers versus non-drinkers, then in the four alcohol unit categories and finally treating alcohol units consumed as a continuous variable. Posterior probability graphs were drawn to assess the probability of the HR exceeding a clinically significant increase, set a priori at a 50% increase in rates of transaminitis, in each of the four categories of alcohol consumption compared with no alcohol consumption. All analyses were carried out for both primary and secondary definitions of transaminitis.

## Results

**Figure 1:**
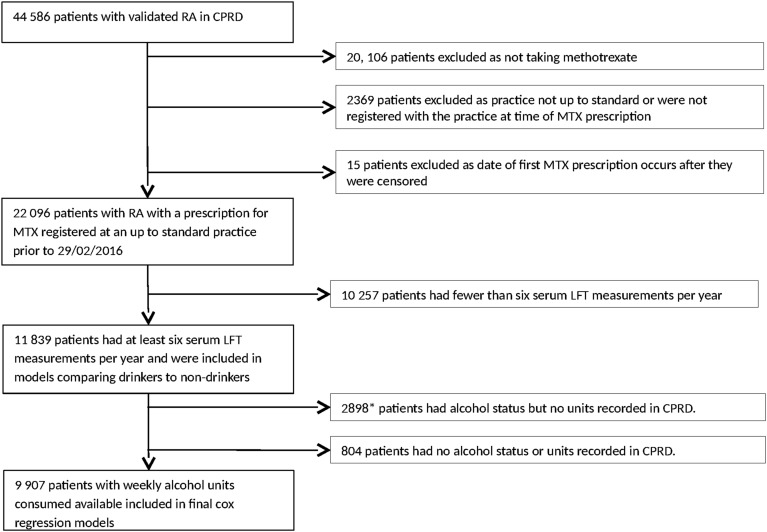
Flow chart of patients included and excluded from the study of the patients with only alcohol status recorded and no weekly units; those who reported drinking no alcohol (1770) were subsequently recorded as drinking zero units per week and were then included in the final model. CPRD, Clinical Practice Research Datalink; LFT, liver function test; MTX, methotrexate; RA, rheumatoid arthritis.

A total of 44 586 patients with RA were identified, of whom 11 839 were included in the study (figure 1, flow chart); 8401 (71%) were female, and mean age (SD) was 61 (13.9) years. Baseline demographic information is shown in table 1; further details of demographics at each stage of exclusion are available in online supplementary table S1. Excluded patients were slightly younger than patients included in the final study. If only information on whether they drank alcohol at all (rather than weekly units) was available, they were more likely to be female, and a much higher percentage drank no alcohol (60% vs 33%). This is likely because these patients would automatically be assumed to drink zero units of alcohol per week, but this may not be recorded separately. As shown in table 1, the vast majority of patients (7764/9907, 78%) were mild drinkers (≤7 units per week) or drank no alcohol; only 799 (8%) consumed more than the UK recommended limit of 14 units per week. Using the primary definition of transaminitis, there were 530 first episodes of transaminitis in 47 090 person-years follow-up, giving a crude event rate of 11.26 per 1000 person-years. Crude rates of transaminitis were similar between patients who consumed any amount of alcohol and non-drinkers, at 10.08 and 10.64 per 1000 person-years, and in the age-adjusted and gender-adjusted Cox model, there was no increased risk in the occurrence of transaminitis in drinkers compared with non-drinkers; HR (95% CI) 1.06 (0.86 to 1.30).

**Table 1:** Baseline demographics

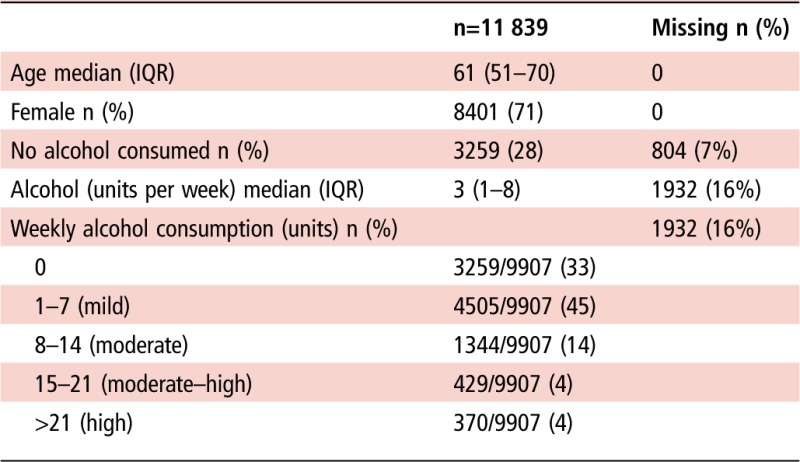

Crude rates of transaminitis appeared to increase with increasing levels of alcohol consumption (table 2). In the adjusted Cox model, mild-to-moderate alcohol consumption (both 1–7 and 8–14 units per week) was not associated with a statistically significant risk of developing transaminitis compared with non-drinkers (table 2), with HRs (95% CIs) of 1.02 (0.82 to 1.28) and 0.98 (0.71 to 1.35), respectively. There was a trend to higher HR with higher levels of alcohol consumption (table 2) and a statistically significant increase in rates of transaminitis for those patients consuming over 21 units per week compared with non-drinkers, both univariately and in the adjusted model; adjusted HR (95% CI) 1.85 (1.17 to 2.93). Finally, when treated as a continuous variable, each increased unit of alcohol consumed was associated with a higher risk of transaminitis; adjusted HR (95% CI) 1.01 (1.00 to 1.02).

**Table 2:** Associations between weekly alcohol consumption and occurrence of transaminitis

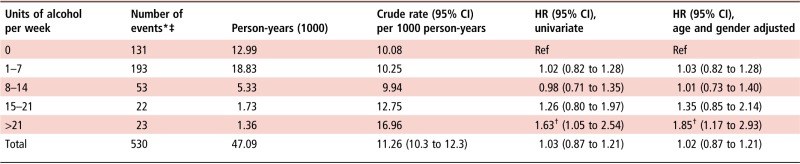

†p<0.01.

*Event=transaminitis, defined as alanine transaminase or aspartate aminotransferase more than three times the upper limit of normal.

‡Not all patients who were defined as drinkers/non-drinkers had alcohol consumption defined in units.

**Figure 2:**
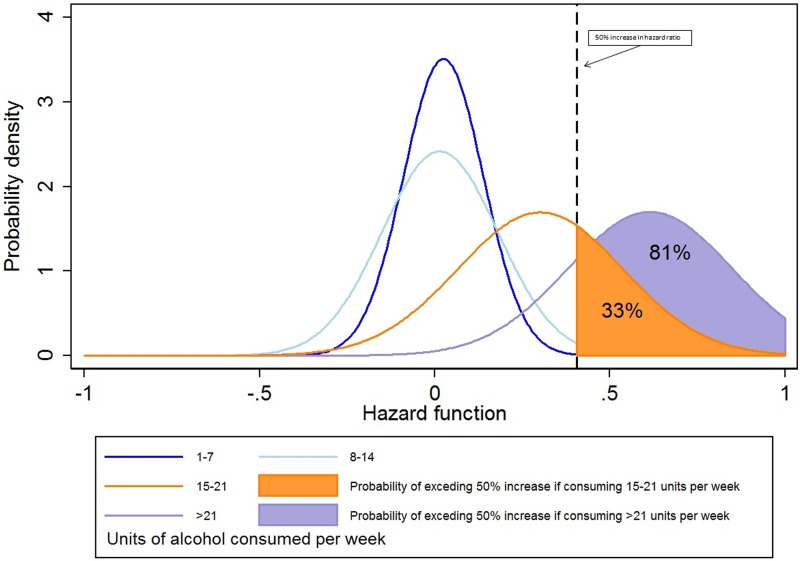
Posterior probabilities of the hazard function. The area under each curve (AUC) represents the probability of the hazard function at that rate of alcohol consumption. The dotted line denotes an arbitrary clinically significant increase in risk of transaminitis of 50% (which would represent an increase in the crude rate from 12 to 18 per 1000 person-years). The AUC to the right of the dotted line is the probability that the hazard function is greater than the clinically significant margin.

Posterior probability graphs (figure 2) demonstrated that alcohol consumption below 14 units per week was associated with a very low probability (0.93%) of having a clinically important (≥50%) increased risk of transaminitis. For alcohol consumption exceeding 14 units per week, the probability of having a clinically important increased risk of transaminitis was higher, specifically 33% and 81% for moderate–high (15–21 units) and high (>21 units) alcohol consumption, respectively.

Using the secondary definition of transaminitis, there was again no increased risk with 1–7 or 8–14 units of alcohol per week (adjusted HR (95% CI) 0.98 (0.83 to 1.16) and 0.95 (0.75 to 1.21), respectively). There was a non-significant increased risk seen in those consuming 15–21 or >21 units of alcohol per week (HRs 1.18 (0.84 to 1.65) and 1.26 (0.87 to 1.81), respectively). The posterior probabilities were lower for all alcohol consumption categories: below 14 units of alcohol per week, the probability of having a clinically important (≥50%) risk of transaminitis was 0.01%, and for moderate–high (15–21 units) and high (>21 units) alcohol consumption, 8% and 17%, respectively (see online supplementary table S2 and figure S1).

10.1136/annrheumdis-2016-210629.supp1 supplementary tables

10.1136/annrheumdis-2016-210629.supp2 supplementary figure

## Discussion

In this study, we have demonstrated that the risk of transaminitis in patients with RA taking MTX does increase with increasing levels of alcohol consumption. However, the risk in those patients who consume ≤14 units of alcohol per week is no greater than those who do not drink alcohol. This is the first study to provide quantifiable estimates of the risk of different levels of alcohol consumption while taking MTX, in a large group of patients who take long-term MTX. The study has important clinical implications. At present, there is uncertainty about the acceptable levels of alcohol consumption while taking MTX, and different rheumatologists and healthcare practitioners may give different advice on what is safe. This can lead to patients avoiding MTX altogether in favour of modest (and perhaps safe) alcohol consumption and thus missing its potential benefits; avoiding any alcohol and potentially affecting their quality of life; or worrying about the potential consequences of any alcohol they consume.

In the literature, there are a small number of studies which have provided a quantitative measure of the risk of alcohol consumption and hepatotoxicity in MTX users.^9^ ^13^ In a study recruiting patients between 1979 and 1990, Malatjalian *et al* 9 examined biopsy before and after MTX therapy retrospectively in a cohort of patients with psoriasis starting MTX. They found no significant difference in progression of liver biopsy grades between patients drinking more or less than 14 units of alcohol per week. Laharie *et al* 13 used a fibroscan technique to investigate the presence of fibrosis in a non-invasive manner in 518 patients taking MTX for a variety of indications, including Crohn's disease and psoriasis as well as RA. They showed that alcohol consumption of >14 units per week was associated with increased fibrosis. However, neither the total dose of MTX nor duration of use was associated with higher fibrosis scores, suggesting the association between alcohol consumption and liver fibrosis is the same in MTX users as in the general population. This study was limited by its cross-sectional nature; in addition, this technique has not yet been adopted widely in clinical practice, and certainly not in the context of monitoring MTX therapy.

A key difficulty with the literature is that studies were often conducted in the 1970s and 1980s, and almost exclusively in patients being treated with MTX for psoriasis, with fewer data on patients with RA.^10^ ^11^ ^20–22^ They were frequently retrospective with small numbers, and some included pretreatment biopsies which demonstrate abnormalities pre-existent to MTX therapy.^10^ Most importantly, however, MTX prescribing and monitoring practices differ markedly now from the time at which the studies were conducted. Critically, liver biopsies are now rarely performed as part of routine monitoring, as there is considerable morbidity and mortality associated with the procedure.^23^ Our data therefore provide more useful insight into the consequences of consuming alcohol while undergoing standard MTX monitoring practice in this era.

Some studies have looked at rates of liver enzyme abnormalities. Curtis *et al* 24 studied patients with both RA and psoriatic arthritis, taking leflunomide and MTX. Their results suggested that LFT derangement in patients taking MTX/leflunomide is significantly more likely in patients who drink one to two alcoholic drinks per day, compared with non-MTX/leflunomide users. However this is not useful if we are attempting to provide information to patients beginning MTX therapy; for them, we need to know the risk of alcohol consumption while taking MTX. Kent *et al* studied^25^ risk factors for the occurrence of abnormal LFTs in a cohort of patients with RA taking MTX from 1991 to 2002. They found no significant association between current alcohol use and abnormal LFTs. However, there are issues with this study. They included any elevation of AST above the ULN as an event of interest, which may capture a large number of false positives of no clinical significance. In addition, they used standard linear regression as opposed to Cox models, which would not take into account the fact that once a person has had an elevated AST, they are more likely to have that blood test repeated. This could lead to counting elevated AST measure more than once, when in fact they are part of the same clinical incidence.

As there is evidence that persistently raised LFTs may be predictive of hepatotoxicity,^17^ we used a secondary transaminitis definition of three consecutive LFTs above the ULN. Using this definition, the results were generally similar with reassurance that up to 14 units of alcohol per week did not increase the risk of hepatotoxicity and a suggestion that higher alcohol consumption did increase the risk. One limitation with this approach is that the outcome definition requires three sequential raised values. If a clinician sees a clinically meaningful rise in LFTs, they would be inclined to stop the MTX therapy following which the transaminases may return to normal and thus not fulfil the outcome definition of three sequential abnormal results. Nonetheless, it is reassuring that both analyses give similar confidence in the safety of modest alcohol consumption.

There are a number of other limitations within our study. The setting within primary care database means that we have to rely on existing general practitioner (GP) codes to identify cases of RA. We used previously validated algorithms^15^; however, it is possible that some misclassification remains. Given that the study design constrained the RA population to MTX users, misclassification is likely to be less than that for an unselected RA cohort. Alcohol use was self-reported, and thus is also prone to misclassification. Patients may be more inclined to underestimate their alcohol consumption, although this would not explain the apparent safety of modest alcohol consumption: were drinkers reporting lower consumption, we would expect hepatotoxicity in these lower alcohol groups to be higher. Validity of self-reported alcohol consumptions in routinely collected clinical data such as CPRD is not well described. However, although response bias has been reported in survey data literature,^26^ there are other data to suggest self-reported alcohol consumption largely valid and reliable, particularly in women.^27^ ^28^ It is possible that alcohol use changed through time following commencement of MTX. Unfortunately, there was not sufficient alcohol data recorded to allow us to consider changing use through time.

Patients were included only if they had six or more LFTs measured per year, as those with fewer blood tests would automatically have a lower chance of abnormal LFTs due to observation bias. That said, patients with high levels of alcohol consumption might be less likely to attend for regular blood test and could have been excluded from the study. However, the baseline characteristics of patients who remained in the study were similar to those who were excluded. Despite the large dataset, the number of events identified was relatively small, particularly in groups consuming high levels of alcohol. Knowing that we might therefore generate results that were not statistically significant, yet still potentially clinically meaningful, we chose to also present results as the probability of the HR exceeding a clinically significant increase of 50%. We demonstrated that this was much higher in patients consuming more alcohol. As with all observational data, there may have been unmeasured confounding that we were not able to adjust for, for example, disease severity. There may have been other comorbidities that could explain the raised LFTs that were not measured. We did not consider the dose of MTX as it was only available in patients included in the study before 2011, as this would have further limited the study power to detect differences between different levels of alcohol consumption. It is possible that hepatotoxicity may be higher in patients with higher MTX dosage, although dose is typically titrated upward while monitoring LFTs. A bias may be possible if clinicians give differing doses to patients who drink different levels of alcohol. At higher levels of alcohol consumption, lower dosage would be more likely, and thus the proven increased risk in the high alcohol groups may well be an underestimate of the true risk. Finally, it should be noted that while we have identified no increased risk of transaminitis when consuming <14 units of alcohol per week, this may not capture all hepatotoxicity. It has been suggested that monitoring LFTs is insufficient to assess long-term damage to the liver from MTX, as patients may progress to fibrosis without ever having episodes of transaminitis.^29^ Nevertheless, serum LFT measurement remains current best practice for monitoring MTX therapy;^7^ therefore, our findings are relevant.

In conclusion, in the largest study of its kind to date, we have shown no increase in the risk of transaminitis in patients who consume <14 units alcohol per week while taking MTX. This may provide the practical and useful information that drinking alcohol within nationally recommended levels in the UK is safe, in terms of risk of transaminitis, for patients commencing MTX therapy for RA. Our study was conducted only in patients with RA and thus cannot be automatically generalisable to other populations. Previous data have suggested that patients with psoriasis may have higher incidence of liver disease in general compared with patients with RA,^13^ and therefore confirmatory studies would be required in these patient groups. Inclusion of acceptable alcohol levels into clinical guidelines and patient information leaflets may well improve informed decision-making, clinical outcomes, reduce decision conflict and improve overall quality of life.
