# Supplementary material for: Quantifying the hepatotoxic risk of alcohol consumption in patients with rheumatoid arthritis taking methotrexate
Source: Ann Rheum Dis. 2017 Mar 23;76(9):1509–14. doi: 10.1136/annrheumdis-2016-210629 (PMC5561375; doi:10.1136/annrheumdis-2016-210629)
Supplement: supplementary tables [file annrheumdis-2016-210629supp001.pdf]

Supplementary table S1: Characteristics of patients excluded from the study

	Reason for exclusion from the study (exclusion occurred sequentially from left to right)						
	**Total RA cohort n=44 586**	**Not taking MTX n=20 106**	**Prescription occurred prior to practice coming up to standard, or before patient joined practice n=2384**	**Fewer than 6 serum LFT measurements per year n=10257**	**No information available on weekly units of alcohol consumed n=2898**	**No information on alcohol consumption (status or units) available n=804**	**Patients included in final model n=9907**
**Age** *mean (sd)*		55 (16.7)	53 (14.6)	58 (14.9)	61 (14.2)	58 (17.2)	61 (13.9)
**Female** *n (%)*		14 138 (70)	1740 (73)	7161 (70)	2314 (80)	578 (72)	8401 (71)
**No alcohol consumed** *n (%)*		3975 (20)	518 (22)	2888 (31)	1770* (61)	n/a	3259 (28)
**Alcohol (units per week)** *mean (sd)*		6.9 (11)	4.8 (8)	6.6 (10)	n/a	n/a	6.2(9.6)

*These 1770 patients were subsequently recoded as consuming zero alcohol units per week and thus are included in the cohort of 9907 patients in the column to the right

Table S2: Associations between weekly alcohol consumption and occurrence of transaminitis (defined using secondary definition).

Units of alcohol per week	Number of events*[$]	Person years (1000)	Crude rate (95% CI) *Per 1000 person years*	Hazard ratio (95% CI) *Univariate*	Hazard ratio (95% CI) *Age & gender adjusted*
0	226	12.56	18.00	ref	ref
1-7	329	18.15	18.12	1.02 (0.86-1.21)	0.98 (0.83-1.16)
8-14	97	5.12	18.95	1.05 (0.83-1.33)	0.95 (0.75-1.21)
15-21	41	1.65	24.82	1.37 (0.98-1.91)	1.18 (0.84-1.65)
>21	36	1.32	27.34	1.48[†] (1.04-2.10)	1.26 (0.87-1.81)
Total	866	45.49	19.04 (17.81-20.35)	1.05 (0.90-1.22)	0.98 (0.84-1.15)

*Event=transaminitis, defined as three consecutive ALT or AST measures greater than ULN

[$]Not all patients who were defined as drinkers/non-drinkers had alcohol consumption defined in units

[†] $p<0.05$
