# Supplementary material for: Quantifying the hepatotoxic risk of alcohol consumption in patients with rheumatoid arthritis taking methotrexate
Source: Ann Rheum Dis. 2017 Mar 23;76(9):1509–14. doi: 10.1136/annrheumdis-2016-210629 (PMC5561375; doi:10.1136/annrheumdis-2016-210629)

Figure S1: Posterior probabilities of the hazard function. The area under each curve (AUC) represents the probability of the hazard function at that rate of alcohol consumption. The dotted line denotes an arbitrary clinically significant increase in risk of transaminitis of 50% (which would represent an increase in the crude rate from 12 to 18 per 1000 person years). The AUC to the right of the dotted line is the probability the hazard function is greater than the clinically significant margin.

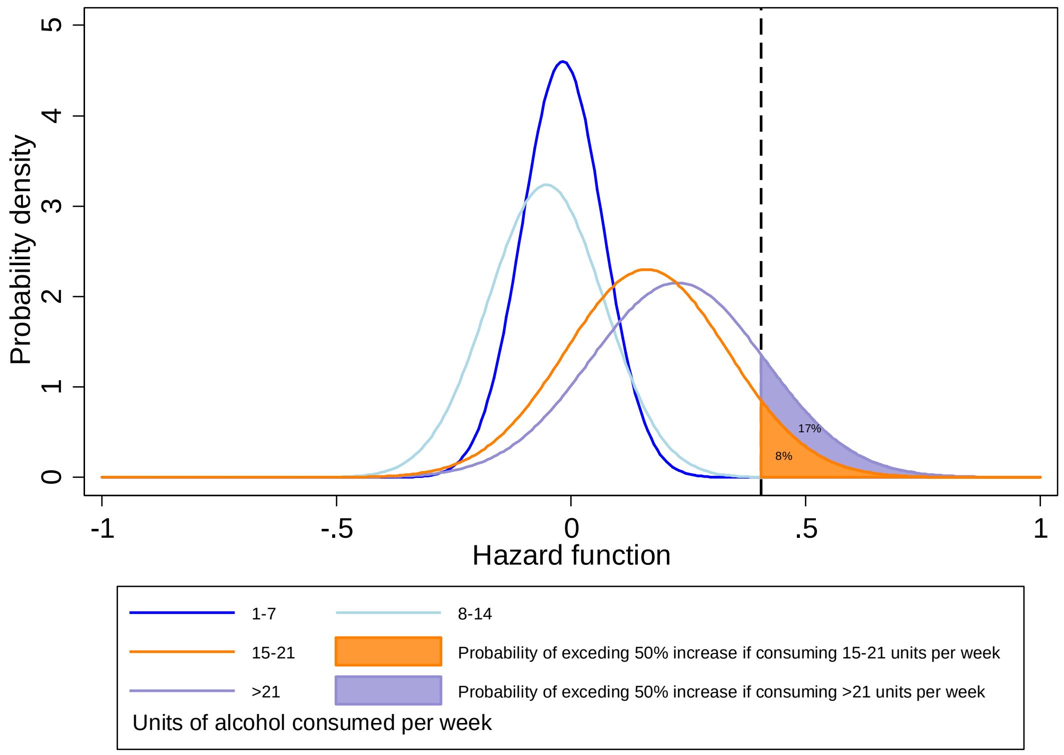

Supplement: supplementary figure [file annrheumdis-2016-210629supp002.pdf]
